# Novel cuproptosis-related long non-coding RNA signature to predict prognosis in prostate carcinoma

**DOI:** 10.1186/s12885-023-10584-0

**Published:** 2023-01-30

**Authors:** Xiaofeng Cheng, Zhenhao Zeng, Heng Yang, Yujun Chen, Yifu Liu, Xiaochen Zhou, Cheng Zhang, Gongxian Wang

**Affiliations:** 1 Department of Urology, Jiangxi Province, The First Affiliated Hospital of Nanchang University, 17 Yongwaizheng Street, Nanchang City, 330000 People’s Republic of China; 2 Jiangxi Institute of Urology, Nanchang City, Jiangxi Province, 330000 China

**Keywords:** Cuproptosis, LncRNA, Prostate carcinoma, Prognostic signature, Machine learning

## Abstract

**Background:**

Cuproptosis, an emerging form of programmed cell death, has recently been identified. However, the association between cuproptosis-related long non-coding RNA (lncRNA) signature and the prognosis in prostate carcinoma remains elusive. This study aims to develop the novel cuproptosis-related lncRNA signature in prostate cancer and explore its latent molecular function.

**Methods:**

RNA-seq data and clinical information were downloaded from the TCGA datasets. Then, cuproptosis-related gene was identified from the previous literature and further applied to screen the cuproptosis-related differentially expressed lncRNAs. Patients were randomly assigned to the training cohort or the validation cohort with a 1:1 ratio. Subsequently, the machine learning algorithms (Lasso and stepwise Cox (direction = both)) were used to construct a novel prognostic signature in the training cohorts, which was validated by the validation and the entire TCGA cohorts. The nomogram base on the lncRNA signature and several clinicopathological traits were constructed to predict the prognosis. Functional enrichment and immune analysis were performed to evaluate its potential mechanism. Furthermore, differences in the landscape of gene mutation, tumour mutational burden (TMB), microsatellite instability (MSI), drug sensitivity between both risk groups were also assessed to explicit their relationships.

**Results:**

The cuproptosis-related lncRNA signature was constructed based on the differentially expressed cuproptosis-related lncRNAs, including AC005790.1, AC011472.4, AC099791.2, AC144450.1, LIPE-AS1, and STPG3-AS1. Kaplan–Meier survival and ROC curves demonstrate that the prognosis signature as an independent risk indicator had excellent potential to predict the prognosis in prostate cancer. The signature was closely associated with age, T stage, N stage, and the Gleason score. Immune analysis shows that the high-risk group was in an immunosuppressive microenvironment. Additionally, the significant difference in landscape of gene mutation, tumour mutational burden, microsatellite instability, and drug sensitivity between both risk groups was observed.

**Conclusions:**

A novel cuproptosis-related lncRNA signature was constructed using machine learning algorithms to predict the prognosis of prostate cancer. It was closely with associated with several common clinical traits, immune cell infiltration, immune-related functions, immune checkpoints, gene mutation, TMB, MSI, and the drug sensitivity, which may be useful to improve the clinical outcome.

**Supplementary Information:**

The online version contains supplementary material available at 10.1186/s12885-023-10584-0.

## Background

Prostate carcinoma (PCa) is the most prevalently diagnosed tumour among men in Western countries and the second leading cause of death [1]. Its incidence shows increases rather than declines in recent years, which accounts for 27% of new cases in males [1]. Androgen-deprivation therapy or combination therapy with other protocols such as radiotherapy and prednisolone initially induces remission in most high-risk non-metastatic PCas but may result in the development of castration-resistant prostate carcinoma (CRPC) [2, 3]. Many patients do not derive great benefit from hormonal therapy due to the heterogeneity of PCa. Additionally, the progression spectrum to CRPC is complicated and variable. Genomic profiling provides the biological feature that would optimize the predictive ability of conventional clinicopathological traits and further improve the clinical outcomes of cancer patients. Indeed, some studies demonstrated that the PI3K/AKT pathway (49%) is the third most frequently mutated, only behind the androgen receptor (AR) (70%) and TP53 (53%) in metastatic CRPC compared to primary tumour, which has improved the understanding and precision treatment of metastatic CRPC [4]. With the advancement of high-throughput sequencing technology long non-coding RNA (LncRNA), as an important gene regulator in mammals and other eukaryotes, has been found to be closely related to tumorigenesis, tumour invasion, metastasis, epithelial mesenchymal transition, and prognosis in PCa [5–7]. Therefore, to improve treatment selection and precision, it is imperative to identify novel lncRNA molecular signatures to predict prognosis and treatment responses in PCa.

Copper, as a catalytic cofactor for essential enzymes, is involved in a variety of critical biochemical pathways [8]. It is closely associated with carcinoma progression and growth, particularly in angiogenesis and metastasis, whose metabolism is also dramatically altered in tumours [9, 10]. Among angiogenesis is critical in the development and progression of PCa, so targeting angiogenesis is a promising treatment strategy for metastatic CRPC [11]. However, several clinical trials demonstrated that it is discouraging outcomes, for instance, bevacizumab, as vascular endothelial growth factor (VEGF)-directed agent, showed superiority in progression-free survival and rates of ≥ 50% prostate-specific antigen (PSA) decrease in patients with chemotherapy-naïve, metastatic CRPC, but it has a higher toxic death rate [11, 12]. Meanwhile, numerous studies demonstrated that copper levels were elevated in various malignancies, particularly in PCa, in which human copper transporter 1 is highly expressed in PCa cells [10, 13]. Therefore, these findings support the use of this element as a target for positron emission tomography imaging in PCa [13]. If the intracellular amount of the copper in mammalian cells exceeds the threshold maintaining homeostatic mechanisms, it would become toxic to cells and trigger cuproptosis [14]. Cuproptosis first described by Tsvetkov et al., which occurs via copper directly binding to the lipoylated components of the tricarboxylic acid (TCA) cycle [14]. This would lead to the accumulation of lipoylated proteins and subsequent loss of iron-sulfur cluster proteins, which in turn triggered proteotoxic stress and ultimately cell death. Growing evidence shows that lncRNAs are associated with epigenetic pathways such as ferroptosis, modification of N6-methyladenosine (m^6^A) methylation in PCa [15–18]. Nevertheless, the role of lncRNA in the biological processes of cuproptosis has not been comprehensively elucidated. Furthermore, novel cuproptosis-related lncRNA signatures for predicting prognosis in PCa remain to be developed.

In this study, cuproptosis-related lncRNA was used to construct a novel consensus signature in training cohorts to assess its predictive value for prognosis and its relationship with the immune microenvironment, immune checkpoints, and several common hormonal therapy drugs, which was validated in the validation and entire The Cancer Genome Atlas (TCGA) cohorts. This work may be contributed to provide novel insights into molecular mechanisms and prognostic prediction in PCa.

## Material and methods

### Data collection and pre-processing

**Fig. 1:**
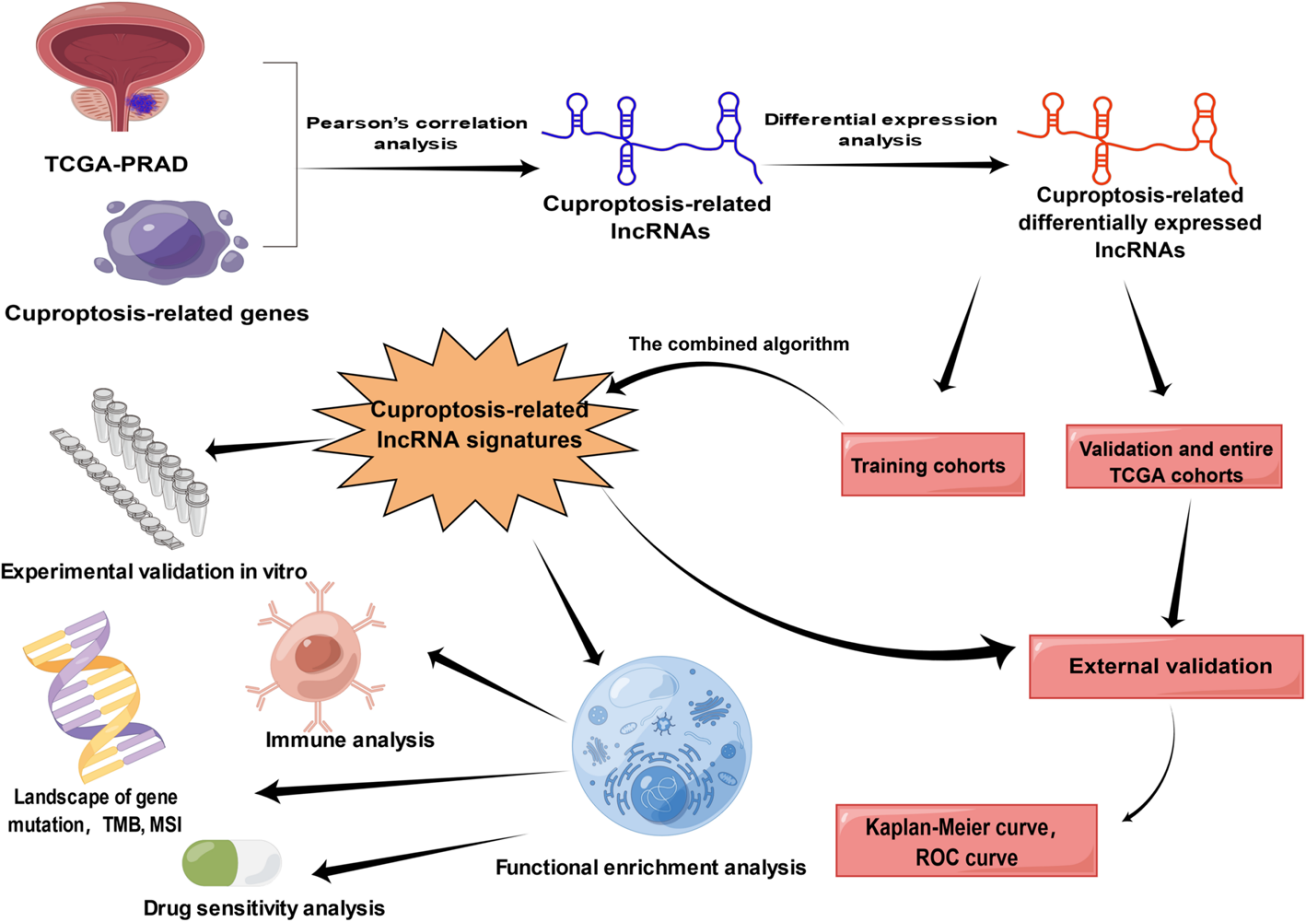
Flow chart. By Figdraw (www.figdraw.com)

The overview of this study is illustrated in Fig. 1. The RNA-seq data (FPKM normalised data) and clinical information of five hundred and fifty-three samples (including 52 non-tumour and 501 tumour tissues) were downloaded from prostate adenocarcinoma (PRAD) of TCGA database (https://portal.gdc.cancer.gov/) in July 2022. The RNA-seq FPKM normalised data would be log-2 transformed for further analysis. Furthermore, missing data including disease-free survival (DFS) and microsatellite instability (MSI) were retrospectively obtained from cBioPortal for cancer genomics database (https://www.cbioportal.org/). Participants with complete DFS information from the TCGA and cBioPortal database were included to develop and validate the stratification signature. The TCGA dataset was randomly divided into training and validation cohorts with a ratio of 1: 1. The 13 cuproptosis-related gene were available from previously published literatures [14], as detailed in supplementary table 1.

### Identification for cuproptosis-related differentially expressed lncRNAs

The correlation between cuproptosis-related gene and lncRNA was assessed via Pearson's correlation analysis. The cuproptosis-related lncRNAs were identified with the Pearson correlation coefficient criteria greater than 0.4 (*R* > 0.4) and P values less than 0.01. Then, cuproptosis-related differentially expressed lncRNAs between normal and PCa tissues were obtained with the criteria of adjusting *p* < 0.05 and | log2 fold change (FC) |> 0.5 via applying the Wilcoxon test in the TCGA cohort.

### Development and validation of cuproptosis‑related lncRNA signatures

Interesting cuproptosis-related lncRNAs that influence PCa DFS were primarily identified through univariate Cox regression based on cuproptosis related differentially expressed lncRNAs. In training cohorts, the algorithm models integrating the least absolute shrinkage and selection operator (Lasso) with the stepwise Cox (direction = both) was used to develop the consensus lncRNA signature via the ‘*glmnet*’ and *‘survival’* package. The risk score was calculated for each patient using the following formula: risk score = ${\sum }_{i=1}^{n}{Coef}_{i}\times {Exp}_{i}$(where *Coef*_*i*_ was the coefficient of selected gene weighted via multivariate Cox regression and *Exp*_*i*_ was the gene expression of selected gene). Each patient was assigned to the high- and low- risk group according to the optimal cutoff value established by the ‘*survminer*’ package. Then, its predictive value was evaluated via the log-rank test and Kaplan–Meier curves, which were generated by using the *‘survival’* and *‘survminer’* packages, respectively. Moreover, the time-dependent receiver operating characteristic (ROC) curve using the 'timeROC' package was also used to evaluate the novel lncRNA signature. Similarly, the abovementioned method also was used to validate the novel risk stratification signature in validation and entire TCGA cohorts.

### Construction of the nomogram and association of the cuproptosis‑related lncRNA signatures with clinicopathological traits

Based on the several available clinical traits and lncRNA signatures, the nomogram was constructed in TCGA cohorts via the *‘rms’* package. To evaluate its predictive performer, Harrell’s concordance index (C-index) was calculated. Subsequently, the time-dependent ROC, as well as 1-year, 3-year, and 5-year calibration curves were also plotted to assess the accuracy and stability of this model, respectively. Then, the association of the cuproptosis‑related lncRNA signatures with common clinical traits also was evaluated, and its predictive value was further assessed by subgroup analysis as well.

### Enrichment analysis

The differentially expressed coding RNA genes between the high- and low-risk groups were uploaded to the Metascape database (http://metascape.org/gp/index.html) to perform gene enrichment analysis [19]. Then, the gene expression matrix and risk group were uploaded to Gene set enrichment analysis (GSEA) software V4.2 for GSEA with permutation = 1000, min size = 15 and max size = 500 to further explore potential pathways.

### Immune cell infiltration, immune-related function, and immune checkpoint analysis

Adaptive or innate infiltrated immune cells were involved in the development, progression, metastasis, and treatment of PCa [20]. In addition, the prevalence of defects in mismatch repair (MMR) in PCa was reported to be between 3 and 5% according to the literature [21]. In the second-line treatment of metastatic CRPC, patients with MMR deficiency were candidates for immune checkpoint inhibitor therapy [22]. Thus, it was vital to assess the status of immune cell infiltration, immune-related function, and immune checkpoints in PCa. The single sample gene set enrichment analysis (ssGSEA) was conducted to assess the amount of 28 immune cell infiltration and immune-related function. The metagenes list of pan-cancer immune was accessible in supplementary table 2 [23]. The proportion of immune cell in both risk group was also evaluated via MCP-counter and TIMER algorithm, which were performed using *‘IOBR’* R package [24]. The key immune checkpoints expression level between both risk groups were also compared to predict the benefit from immunotherapy.

### Landscape of gene mutation, tumour mutational burden, microsatellite instability, and drug sensitivity analysis

The mutation frequencies and oncoplot waterfall plots for both risk groups were analysed and visualized with the *‘maftools’* package.

Tumour mutational burden (TMB) and microsatellite instability (MSI) could be regarded as an indicator to predict immunotherapy response. The presence of homologous recombination deficiencies, such as BRCA1/2 mutations, can lead to TMB amplification and contribute to immune checkpoint inhibitor sensitivity [25]. Previous studies also showed that TMB was closely related to common clinical traits such as T and N stage and patients with high TMB had worse survival than those with low TMB in PCa [26]. 45.5% of patients with MSI-high/ or mismatch repair–deficient metastatic CRPC derived durable clinical benefit from immune check-point blockade [27].Thus, it is essential to assess the status of TMB and MSI and their relationship with the cuproptosis‑related lncRNA signatures in both risk group.

Several common drug sensitivities were estimated from the Genomics of Drug Sensitivity in Cancer database (https://www.cancerrxgene.org/) via the *‘pRRophetic’* package [28].

### Validation of the cuproptosis‑related lncRNA signature

Trizol reagent (ComWin Biotech, Beijing, China) and reversed transcribed into cDNA with the TransScript First-Strand cDNA Synthesis SuperMix kit (TransGen Biotech, Beijing, China) was used to extract total RNA from prostate normal or cancer cell lines RWPE-1, PC3, DU145, VCaP, and LNCaP according to the manufacturer’s description. Real-time quantitative polymerase chain reaction (RT-qPCR) was conducted in triplicate with qPCR SYBR Green SuperMix (TransGen Biotech, Beijing, China). β-Actin was employed as an internal reference gene to normalize relative expressions of lncRNA with the 2^−ΔΔCT^ method. The primer sequences were accessible in supplementary Table 3.

### Statistical analysis

All data processing and statistical analyzes were performed using R software (Version 4.2.1). The Wilcoxon test or independent t test was used to analyse continuous data, while the Chi-square test or Fisher's exact test categorical data. A *P*- value less than 0.05 was considered statistically significant criteria.

## Results

### Identification of cuproptosis-related differentially expressed lncRNAs

**Fig. 2:**
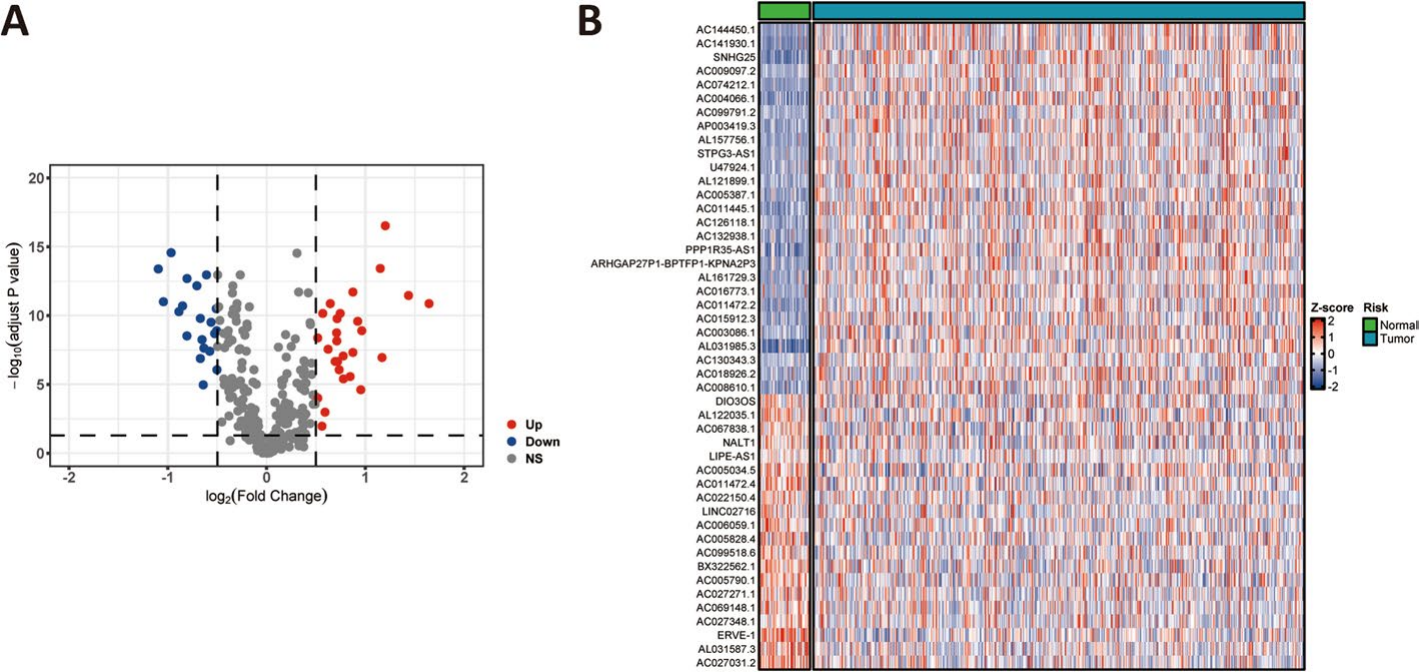
Cuproptosis-related differentially expressed lncRNAs in TCGA cohorts.** A** Volcano plots of cuproptosis-related differentially expressed lncRNAs. **B** Heatmap plots of cuproptosis-related differentially expressed lncRNAs

Thirteen cuproptosis-related genes were available from the study of Tsvetkov P et al. [14]. The result of Pearson’s correlation analysis demonstrated that 311 lncRNAs expression level were tightly correlated with the cuproptosis-related gene, which was illustrated in supplementary Table 4. Forty-seven differentially expressed lncRNAs were identified via the Wilcoxon test in the TCGA cohort, which contained 20 down-regulated genes and 27 up-regulated lncRNA genes, as shown in Figs. 2A and B. Then, the TCGA cohorts were assigned to the training and validation cohorts. The baseline characteristics of PCa patients between both cohorts are shown in Table 1.

**Table 1:** Clinical characteristics in the training and validation cohorts

Variable	All cohorts (*N* = 489)	Training cohorts (*N* = 244)	Validation cohorts (*N* = 245)	*P*-value
**Age: n (%)**				0.276
< 61	221 (45.2%)	104 (42.6%)	117 (47.8%)	
≥ 61	268 (54.8%)	140 (57.4%)	128 (52.2%)	
**Race: n (%)**				0.589
American indian or Alaska native	1 (0.2%)	1 (0.4%)	-	
Asian	12 (2.5%)	4 (1.6%)	8 (3.3%)	
Black or african american	57 (11.7%)	29 (11.9%)	28 (11.4%)	
White	406 (83.0%)	202 (82.8%)	204 (83.3%)	
Not reported	13 (2.7%)	8 (3.3%)	5 (2.0%)	
**M stage: n (%)**				0.558
M0	447 (91.4%)	223 (91.4%)	224 (91.4%)	
M1	2 (0.4%)	2 (0.8%)	-	
Mx	40 (8.2%)	19 (7.8%)	21 (8.6%)	
**N stage: n (%)**				0.841
N0	340 (69.5%)	169 (69.3%)	171 (69.8%)	
N1	79 (16.2%)	38 (15.6%)	41 (16.7%)	
Nx	70 (14.3%)	37 (15.2%)	33 (13.5%)	
**T stage: n (%)**				0.916
T2	187 (38.2%)	95 (38.9%)	92 (37.6%)	
T3	291 (59.5%)	144 (59.0%)	147 (60.0%)	
T4	11 (2.2%)	5 (2.0%)	6 (2.4%)	
**Gleason score: n (%)**				0.762
6	45 (9.2%)	26 (10.7%)	19 (7.8%)	
7	243 (49.7%)	120 (49.2%)	123 (50.2%)	
8	63 (12.9%)	32 (13.1%)	31 (12.7%)	
9	135 (27.6%)	64 (26.2%)	71 (29.0%)	
10	3 (0.6%)	2 (0.8%)	1 (0.4%)	

### Development and validation of cuproptosis‑related lncRNA signatures

**Fig. 3:**
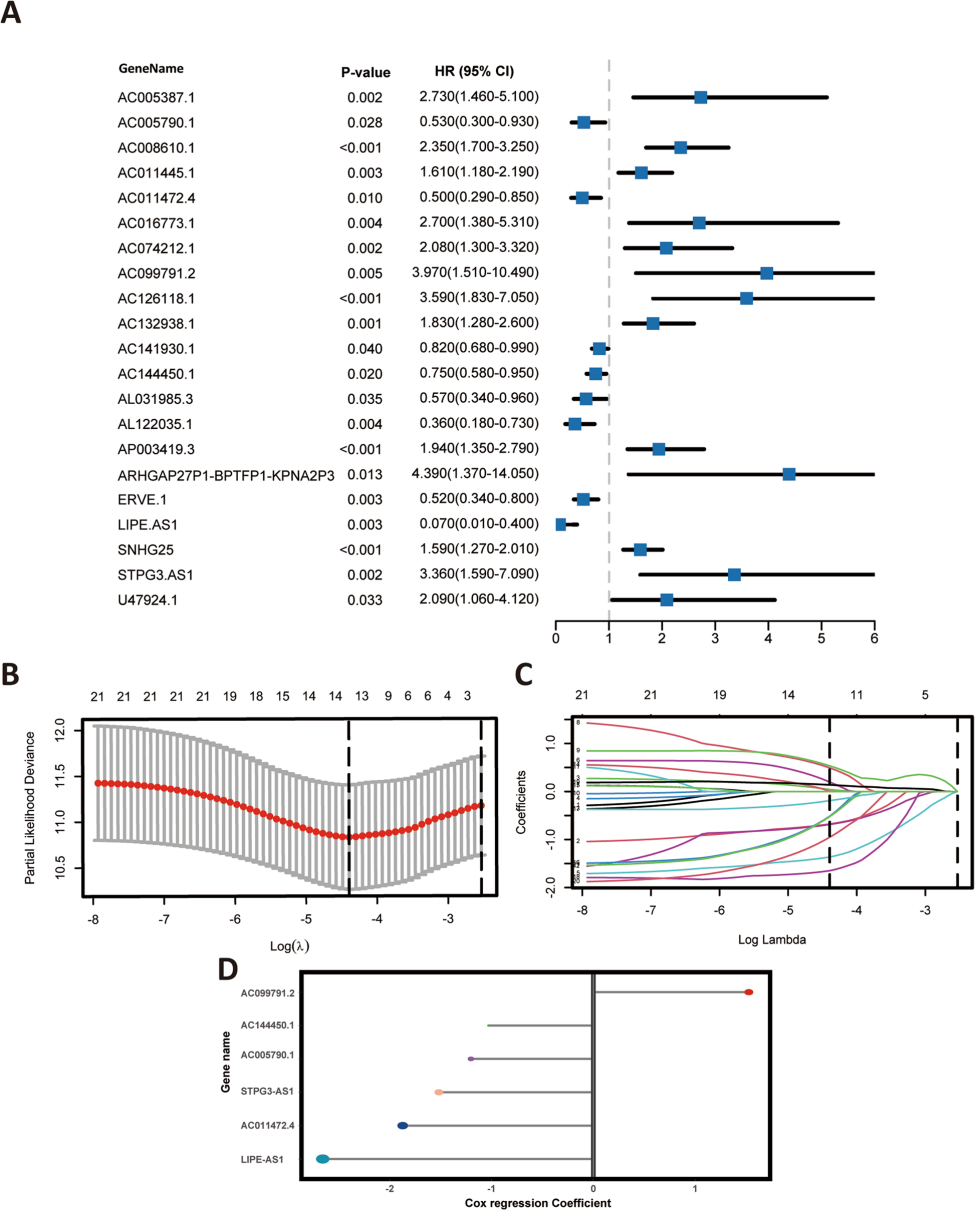
Regression analysis.** A** Univariate Cox regression analysis of cuproptosis-related differentially expressed lncRNAs; (**B, C**) Lasso regression analysis of AAM‐related DEGs; (**D**) Coefficients of 6 lncRNAs obtained in stepwise Cox regression

In the training cohorts, univariate Cox regression based on cuproptosis-related differentially expressed lncRNAs was ultimately selected for the 21 prognostic lncRNAs, including AC005387.1, AC005790.1, AC008610.1, AC011445.1, AC011472.4, AC016773.1, AC074212.1, AC099791.2, AC126118.1, AC132938.1, AC141930.1, AC144450.1, AL031985.3, AL122035.1, AP003419.3, ARHGAP27P1-BPTFP1-KPNA2P3, ERVE.1, LIPE-AS1, SNHG25, STPG3-AS1, U47924.1, as detailed in Fig. 3A. Then, the combined algorithm models of Lasso and the stepwise Cox (direction = both) were used to construct the prognostic signature in order to effectively avoid multicollinearity among various variables and to reduce data dimensionality. When the optimal lambda was 0.0124, the partial likelihood of deviance reached the minimum. Thus, AC005790.1, AC008610.1, AC011472.4, AC016773.1, AC099791.2, AC126118.1, AC144450.1, AL122035.1, ARHGAP27P1-BPTFP1-KPNA2P3, ERVE.1, LIPE-AS1, SNHG25, STPG3-AS1, and U47924.1 were selected (Fig. 3B and C). The stepwise Cox (direction = both) was applied to further screen key lncRNAs based on the Lasso regression with tenfold cross-validation. Eventually, the risk score of each patient was calculated with the following formula: risk score = AC005790.1*-1.202 + AC011472.4*-1.872 + AC099791.2*1.528 + AC144450.1*-1.027 + LIPE-AS1*-2.658 + STPG3-AS1*-1.517 (Fig. 3D). Then, all patients in the TCGA cohorts were assigned to the low- and high-risk group according to the optimal cut-off value for this prognostic signature.

**Fig. 4:**
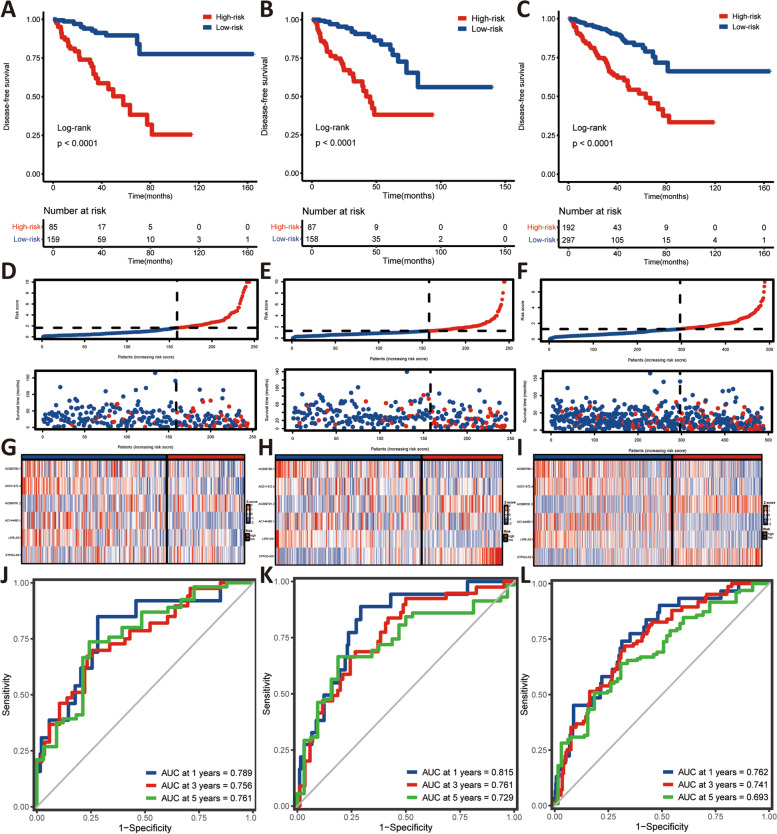
The prognostic predictive performance of cuproptosis-related lncRNA signature in PCa. **A-C** Kaplan–Meier survival analysis between both risk groups in training (**A**), validation (**B**), and entire TCGA (**C**) cohorts; (**D-F**) The trend in survival status with increasing risk scores in training (**D**), validation (**E**), and entire TCGA (**F**) cohorts; (**G-I)** Heatmap plots of the individual prognostic lncRNAs in training (**G**), validation (**H**), and entire TCGA (**I**) cohorts; (**J-L**) Time-independent receiver operating characteristic (ROC) curve of this prognostic signature in training (**J**), validation (**K**), and entire TCGA (**L**) cohorts

Kaplan–Meier (K-M) survival curve analysis demonstrated that DFS in the high-risk group was apparently shorter than in the low-risk group in the training dataset (Fig. 4A), which was validated by the validation (Fig. 4B) and entire TCGA dataset (Fig. 4C). Consistently, the risk score distribution and survival status of both risk group was evaluated via the risk score formula in the training (Fig. 4D), the validation (Fig. 4E), and entire TCGA dataset (Fig. 4F), respectively. The expression heatmaps of the six selected cuproptosis-related lncRNAs for the training datasets were shown in Fig. 4G, the validation datasets in Fig. 4H, and the entire TCGA datasets in Fig. 4I, respectively. ROC analysis of DFS was conducted to assess the discrimination of the cuproptosis-related lncRNA signature. Its results suggested that the 1-, 3-, and 5-year areas under the ROC curves (AUCs) were 0.789, 0.756, and 0.761 in the training cohorts (Fig. 4J), 0.815, 0.761, and 0.729 in the validation cohorts (Fig. 4K), as well as 0.762, 0.741, and 0.693 in the entire TCGA cohorts (Fig. 4L), respectively.

### Construction of a nomogram

**Fig. 5:**
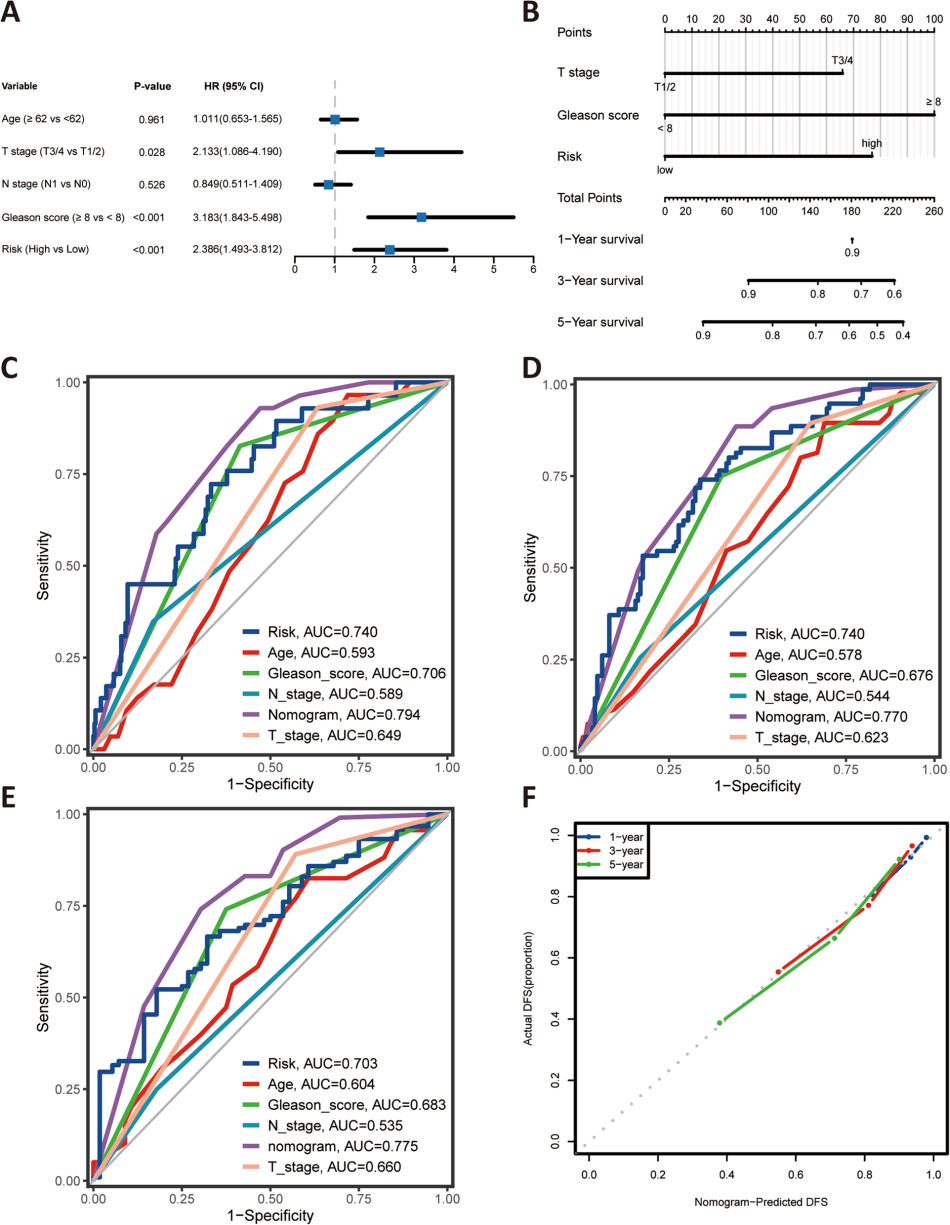
Multivariate cox regression analysis and prognostic predictive performance of the nomogram, risk (cuproptosis-related lncRNA signature), and other clinical indicators. **A** Multivariate Cox regression analysis; (**B**) Nomogram for predicting 1-, 3-, and 5-year DFS. **C-E** ROC curves for 1-year (**C**), 3-year (**D**), and 5-year (**E**) DFS based on the nomogram, risk, and other clinical indicators, respectively; (**F**) the calibration plots for predicting 1-, 3-,5-year DFS of the nomogram, respectively

After rigorous screening of patients with complete and definitive clinicopathological information, four hundred and twenty PC patients were ultimately included for further analysis in the entire TCGA cohort. The results of the multivariate Cox regression showed that T stage, Gleason score, and risk were considered as independent prognostic metrics, as detailed in Fig. 5A. Thus, the nomogram was constructed to predict 1-, 3-, and 5-year DFS based on this multivariate Cox regression in TCGA cohort (Fig. 5B). To evaluate the accuracy of this model, the ROC curves were utilised to compare this cuproptosis-related LncRNA signature with several available clinical traits (Fig. 5C-E). This result showed that the predictive value of this nomogram model was more optimal compared to several clinical traits such as T stage, N stage, and Gleason score at 1, 3, and 5 years. Subsequently, the calibration curves for the nomogram indicated that the actual DFS was well consistent with the predicted DFS (Fig. 5F).

### Relationship between the lncRNA signature and clinicopathological traits

**Fig. 6:**
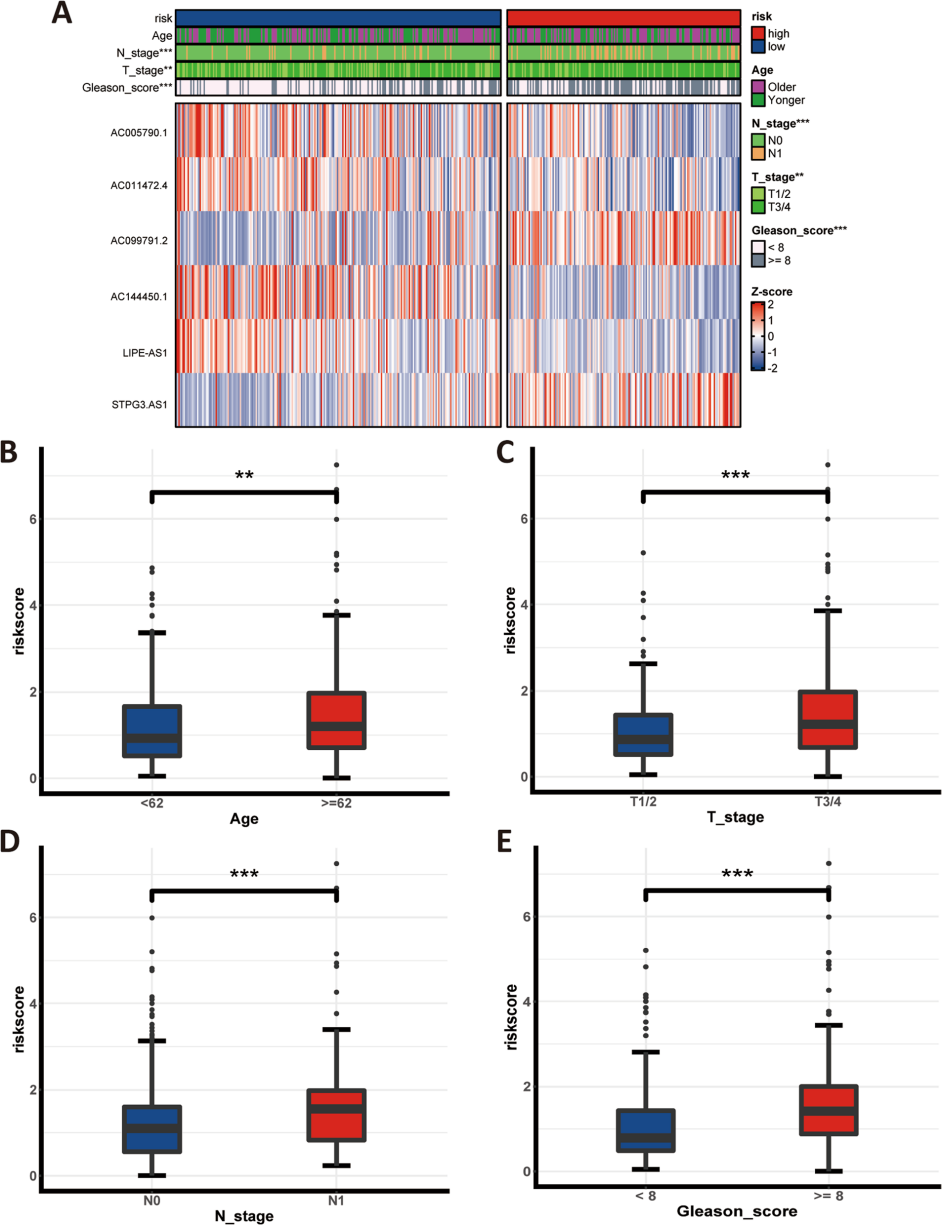
Association between the cuproptosis-related lncRNA signature and clinical traits. **A** Heatmap plot of individual prognostic lncRNA in cuproptosis-related signature and correlation between it and other clinicopathological traits; (**B-E**) Box plots of risk scores for different stratification subgroups; ns: *p* > 0.05; *: *p* < 0.05; **: *p* < 0.01; ***: *p* < 0.001

**Fig. 7:**
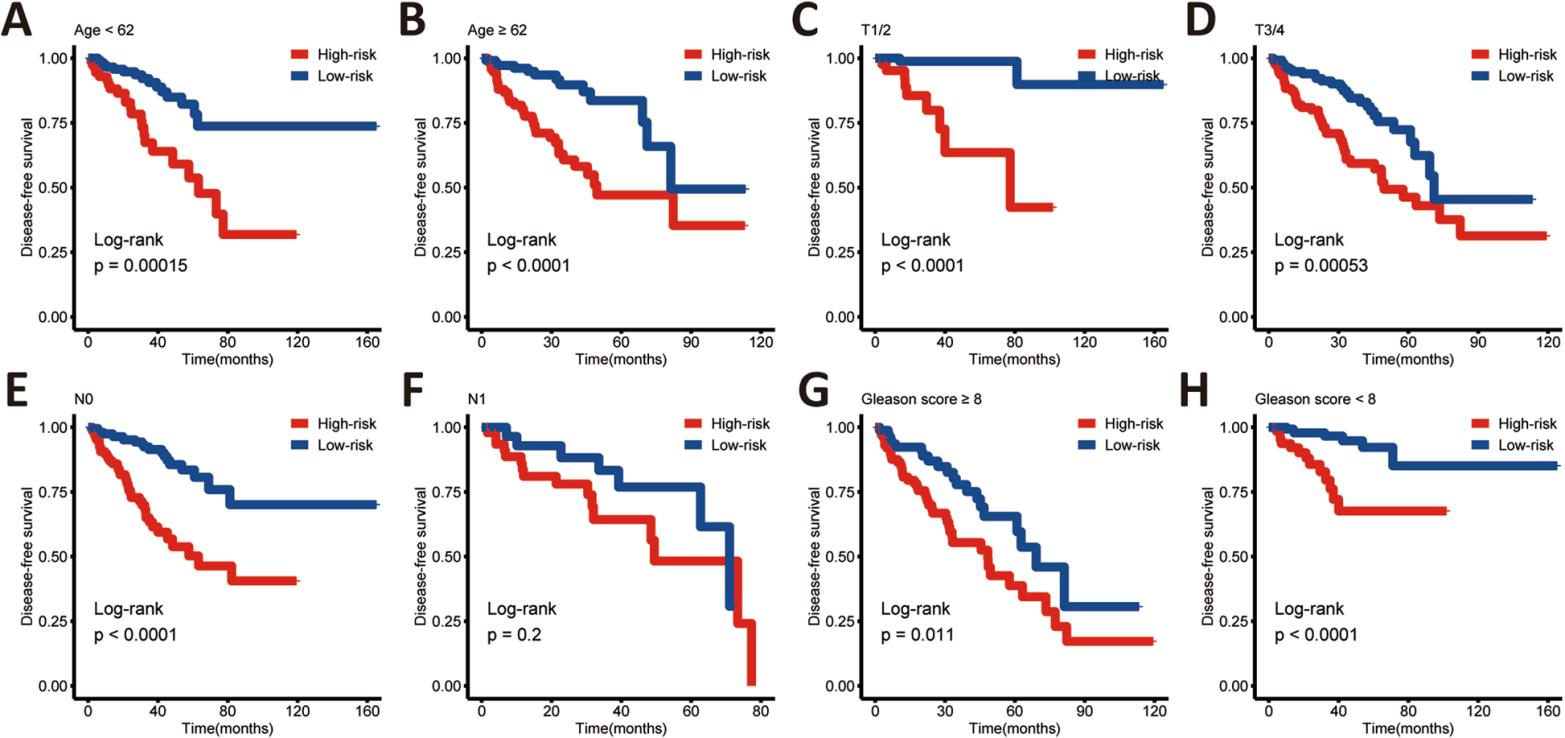
Subgroup analysis of the cuproptosis-related lncRNA signature **(A-H)**

In the cuproptosis-related lncRNA signature, the expression level of AC099791.2 and STPG3-AS1 were upregulated in the high-risk group, whilst AC005790.1, AC011472.4, AC144450.1, and LIPE-AS1 were downregulated in the high-risk group (Fig. 6A). To further analyse the relationship between the prognostic lncRNA signature and clinicopathological traits, the risk score was compared in different cohorts, showing that the PCa patient with the older, advanced T and N stage, as well as the worse Gleason score had the higher risk score (Fig. 6B-E). Moreover, to evaluate the prognostic predictive value of the lncRNA signature in different stratified cohorts, the subgroup analysis was performed, indicating that it provides a reliable and accurate prediction ability in patients with age < 62, age ≥ 62, T1/2, T3/4, N0, N1, Gleason score ≥ 8, and Gleason score < 8 (Fig. 7A-H). In sum, the cuproptosis-related lncRNA signature was closely associated with several common clinicopathological traits and has surprising potential for predicting prognosis in PCa patients.

### Functional enrichment analysis

**Fig. 8:**
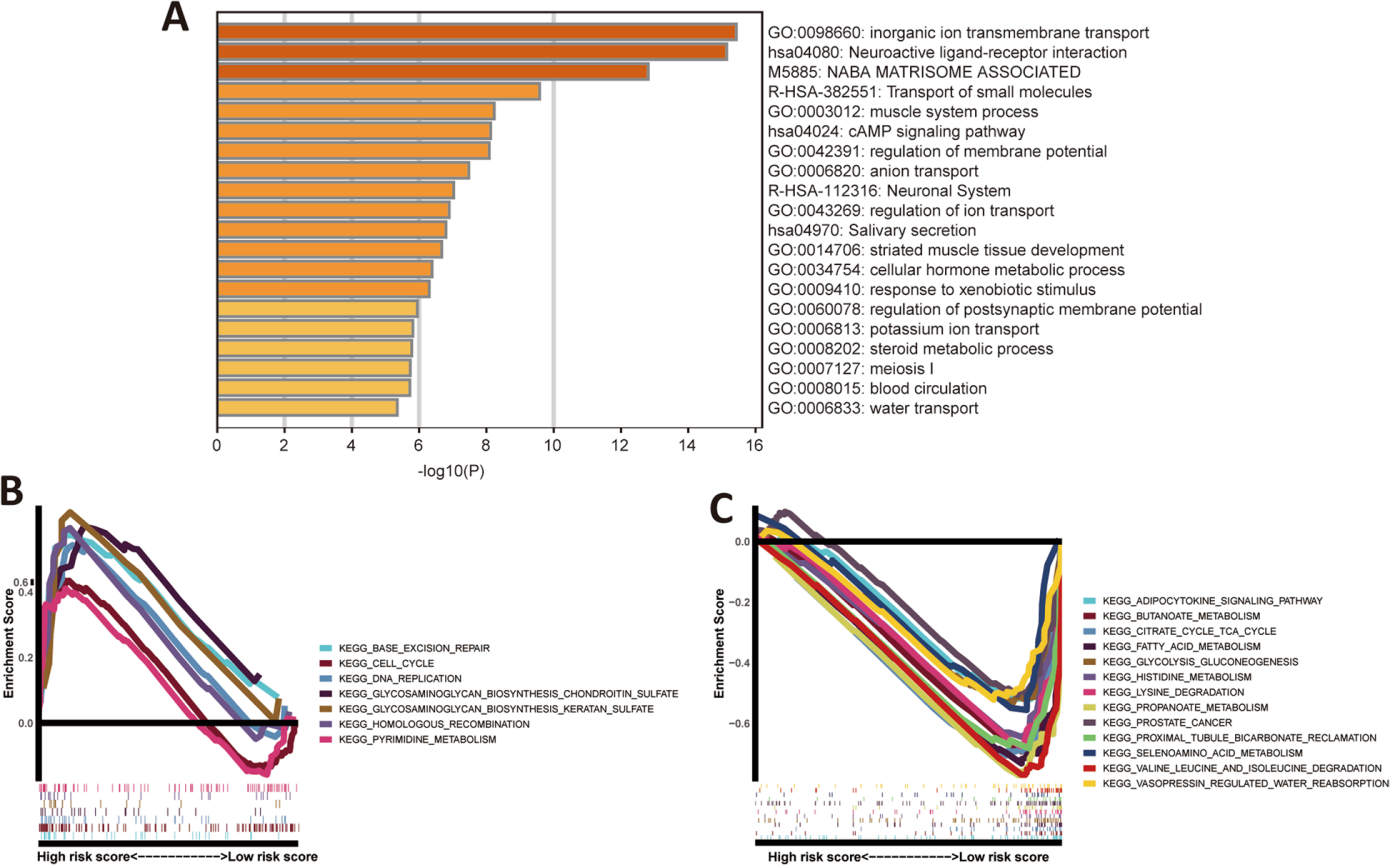
Functional enrichment analysis. **A** Enrichment analysis based on the Metascape database; (**B-C**) Gene set enrichment analysis in the high-risk group (**B**) and the low-risk group (**C**)

Nine hundred ninety-one differentially expressed coding RNA genes between the high- and low-risk groups were acquired with cut-off criteria of adjusting *p* < 0.05 and |log2FC|≥ 0.5 and then were uploaded to Metascape database to explore the potential cellular functions and processes. As shown in Fig. 8A, these genes were mainly enriched in ion and small molecules transport. Meanwhile, GSEA was conducted to investigate the signalling pathways underlying the risk signature, suggesting that the high-risk group was mainly enriched in base excision repair, cell cycle, DNA replication, and so on (Fig. 8B). Likewise, the low-risk group mainly focused on the adipocytokine signaling pathway, the citrate cycle TCA cycle, fatty acid metabolism, prostate cancer, etc. (Fig. 8C).

### Immune analysis

**Fig. 9:**
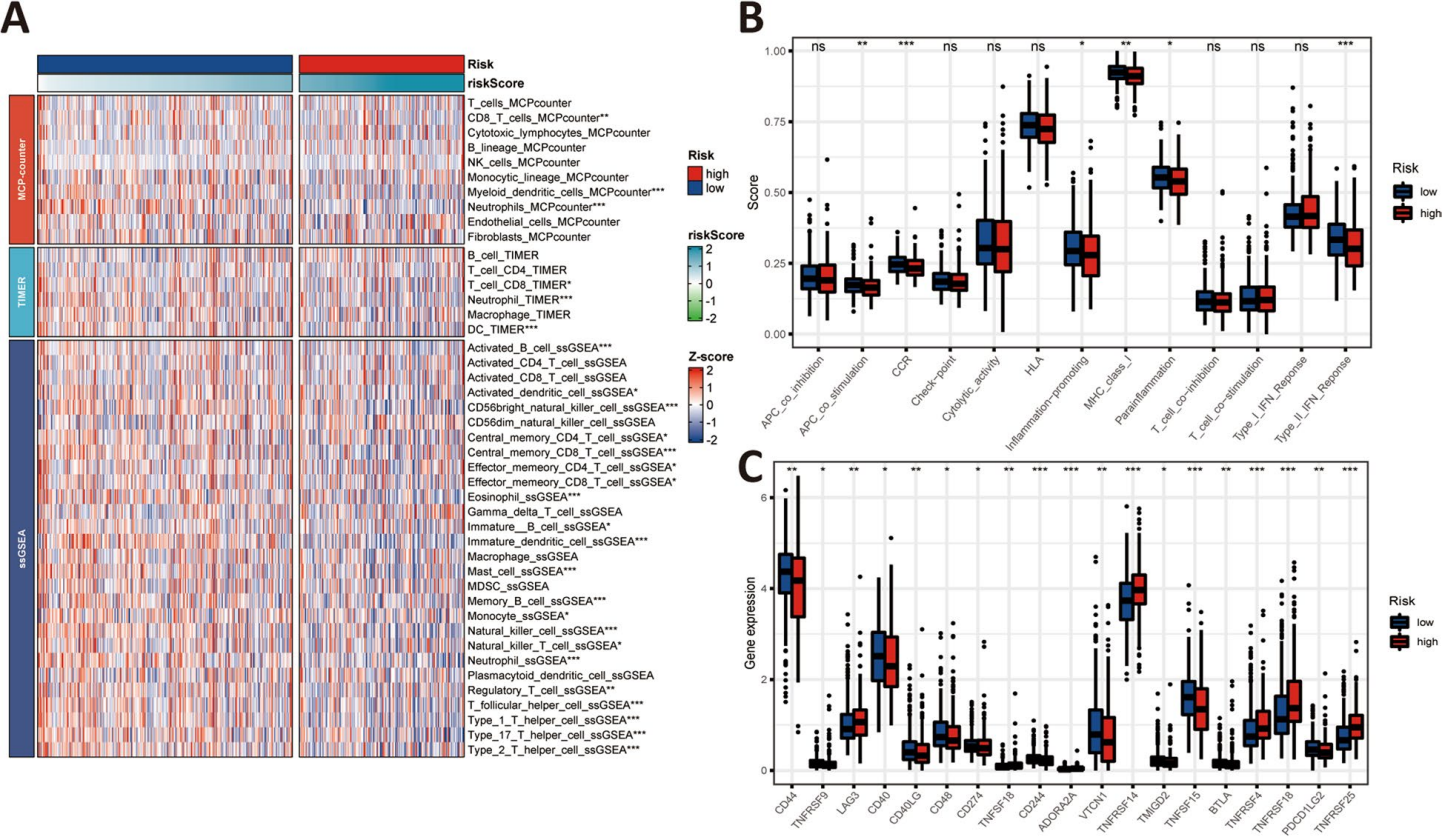
Immune infiltration, immune status, and immune checkpoints analysis. **A** The heatmap of immune cells content in both risk group via MCP-counter, TIMER, and ssGSEA algorithm, respectively; (**B**) The different immune status in both risk group via ssGSEA algorithm; (**C**) Gene differential expression analysis of key immune checkpoints; ns: *p* > 0.05; *: *p* < 0.05; **: *p* < 0.01; ***: *p* < 0.001

Immune cell infiltration, immune-related function, and immune checkpoints were evaluated to explore the effect of immune in the development, progression, and metastasis of PCa. According to 28 immune cells infiltration assessed by ssGSEA, the result demonstrated that the high-risk group trend to have an immunosuppressive microenvironment (Fig. 9A). In MCP-counter algorithm, the content of CD8 T cells, myeloid dendritic cells, and neutrophils were lower in the high-risk group than that in the low-risk group. In the TIMER algorithm, CD8 T cells, neutrophils, and DCs had a higher ratio in the high-risk group. Similarly, in the ssGSEA algorithm, the proportion of the remaining cells was higher in the low-risk group than in the high-risk group, except for activated CD4 and CD8 T cells, CD56dim natural killer cell, Gamma delta T cell, macrophage, myeloid-derived suppressor cell (MDSC), and plasmacytoid dendritic cell (Fig. 9A). Likewise, the immune-related function was evaluated, demonstrating that the level of antigen presenting cell (APC) co-stimulation, co-stimulation C–C chemokines receptors (CCR), inflammation-promoting, major histocompatibility complex (MHC) class I, parainflammation, type II interferon (IFN) reponse was lower in the high-risk group than in the low-risk group (Fig. 9B). As illustrated in Fig. 9C, the results of the differential expression analysis of the key immune checkpoints in both risk group indicated that the expression level of CD44, TNFRSF9, CD40, CD40LG, CD48, CD274 (PD-L1), CD244, VTCN1, TMIGD2, TNFSF15, BTLA, and PDCD1LG2 (PD-L2) were downregulated in the high-risk group. On the contrary, few immune checkpoints, such as LAG3, TNFSF18, ADORA2A, TNFRSF14, TNFRSF18, and TNFRSF25 were upregulated.

### Landscape of gene mutation, tumour mutational burden, microsatellite instability, and drug sensitivity analysis

**Fig. 10:**
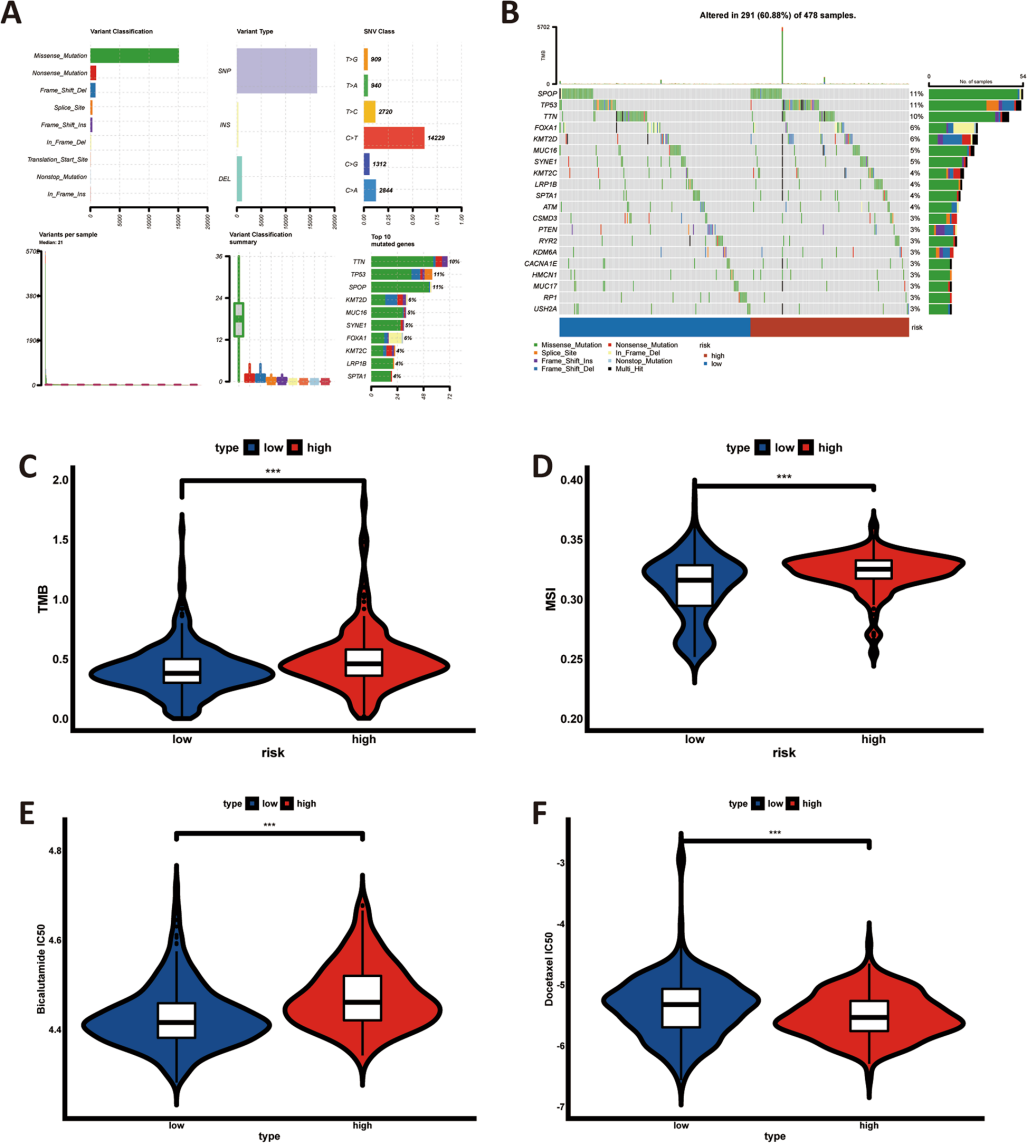
Gene mutation, tumour mutational burden and microsatellite instability, along with drug sensitivities analyses. (**A-B**) Landscape of gene mutation in PCa (**A**) and both risk group (**B**), respectively; (**C-D**) Association between the cuproptosis-related lncRNA signature and tumour mutational burden (**C**) as well as microsatellite instability (**D**); (**E–F**) Drug sensitivities analyses of bicalutamide (**E**) and docetaxel (**F**) in both risk group, ns: *p* > 0.05; *: *p* < 0.05; **: *p* < 0.01; ***: *p* < 0.001

The miscellaneous mutation was the most common variant classification in PCa, followed by the nonsense mutation (Fig. 10A). Single nucleotide polymorphisms were the most common variant type, and C > T ranked as the top SNV class (Fig. 10A). As shown in Fig. 10B, 291 of 478 (60.88%) PCa samples had genetic mutations in both risk groups. The SPOP and TP53 gene had the highest mutation frequency (11%), followed by TNN (10%), FOXA1 (6%), and KMT2D (6%).

TMB and MSI, as one of the metrics to predict the benefit of immunotherapy, were utilized to guide clinical practice. To explore the relationship between them with the risk signature, the TMB and MSI score in both risk group were compared. It indicated that the high-risk group had higher TMB and MSI score (*p* < 0.001), as detailed in Fig. 10C-D.

Bicalutamide and docetaxel were regarded to be the first-line drug in PCa treatment regimens. Thus, their half-maximal inhibitory concentration (IC_50_) was compared as an indicator of drug sensitivity via the *‘pRRophetic’* package. PCa patients in the high-risk group were more sensitive to bicalutamide than in the low-risk group, in contrast to docetaxel (Fig. 10E-F).

### Validation of cuproptosis‑related lncRNA gene

**Fig. 11:**
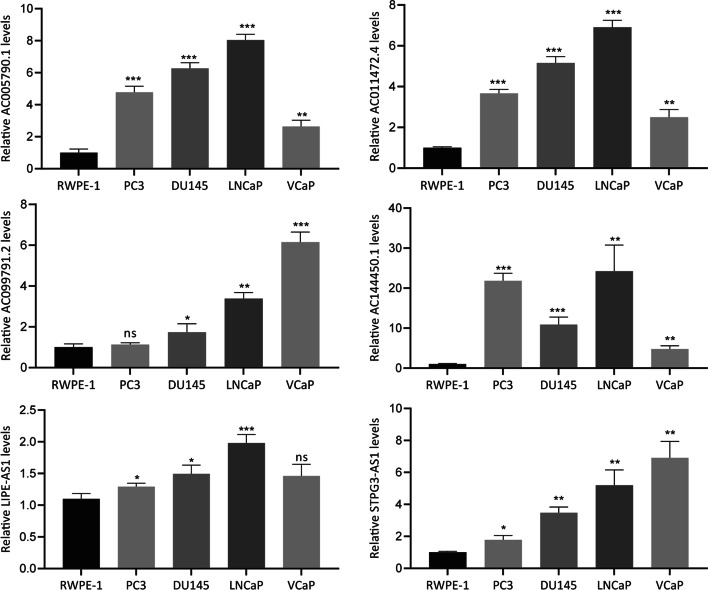
Validation of the expression levels of cuproptosis-related prognostic lncRNAs via RT-qPCR; ns: *p* > 0.05; *: *p* < 0.05; **: *p* < 0.01; ***: *p* < 0.001

The level of expression of lncRNA was measured in prostate normal or cancer cell lines by RT-qPCR, showing that the expression of AC005790.1, AC011472.4, AC144450.1, and STPG3-AS1 was up-regulated in PCa cell lines compared to RWPE1. The expression level of AC099791.2, and LIPE-AS1 were slightly elevated in corresponding cancer cell lines (Fig. 11).

## Discussion

LncRNA has mRNA-like transcripts which are not capable of encoding proteins or peptides. Its plays an essential role in the regulation of gene expression at the transcriptional, translational, and post-translational levels [29]. Meanwhile, cuproptosis, as an important cellular function and process, has recently been discovered [14]. Copper homeostasis plays an important role in the development of various tumours, an imbalance of which can lead to cytotoxicity and further affect cancer cell growth and proliferation [30]. Tsvetkov found that cuproptosis was triggered by copper-induced aggregation of lipid-acylated proteins and loss of iron-sulfur (Fe-S) cluster proteins, as well as increased proteotoxic stress through direct binding of lipid-acylated components of the tricarboxylic acid (TCA) cycle [14]. Given that lipid acylation and Fe-S cluster proteins are widely and conservatively present in nature, targeted therapy based on this mechanism of cuproptosis may be promising options in cancer. Therefore, it is essential to construct and develop the gene signature to observe the extent of cuproptosis in tumours. In addition, due to the heterogeneity of PCa, their prognosis and treatment outcomes were highly variable and complicated. However, the insufficiency of a conventional clinical management tool could sometimes lead to overtreatment or undertreatment of PCa patients. For example, androgen receptor splice variant 7 (AR-V7) could precisely identify who would benefit from treatment with novel androgen receptor blocking agents in metastatic CRPC, thus it might be a reliable prognostic biomarker [31]. However, it lacks the predictive performer for resistance to taxanes [31]. To bridge this gap, the novel robust prognostic lncRNA signature was developed to more accurately predict PCa survival, immune infiltration feature, and drug benefits in this study.

In our study, Pearson's correlation and differential expression analysis was applied to identify differentially expressed cuproptosis-related lncRNAs. The novel lncRNA signature which consist of AC005790.1, AC011472.4, AC099791.2, AC144450.1, LIPE-AS1, and STPG3-AS1 was further developed via the combined algorithm [Lasso and stepwise Cox (direction = both)]. This model can not only effectively avoid the issue of multicollinearity but also precisely screen the key variables. Both the K-M survival curve and the ROC curve presented that the consensus lncRNA signature could stratify the risk of PCa patients with high precision and stability performance in training, validation, and entire cohorts of TCGA. Moreover, the cuproptosis-related lncRNA signature was also closely associated with the common clinical feature, such as T stage, N stage, and Gleason score. To better understand the mechanism underlying the cuproptosis-related lncRNA signature, it is necessary to analyse the role of each gene in cuproptosis. Chu et al. founded that the Toll-like receptors related prognostic gene signature involving AC011472.4 is an independent risk indicator in colorectal cancer [32]. Liu et al. also demonstrated that the new immune-related lncRNA based on LIPE-AS1 can independently assess the prognosis of patients with cervical squamous cell carcinoma [33]. Similarly, Li et al. presented that ferroptosis-related lncRNA consisting of fifteen including STPG3-AS1 was closely associated with the prognosis of colorectal cancer. The studies mentioned above all showed that the cuproptosis-related lncRNA signature gene was involved in the development and prognosis of the tumour.

To further explore the potential mechanism of the cuproptosis-related lncRNA signature in PCa, these functional analyzes, including functional enrichment, immune, gene mutation, tumour mutational burden, microsatellite instability, and drug sensitivity analysis, were conducted. The results of the functional enrichment analysis found that the differentially expressed coding RNA genes between both groups were mainly enriched in the transport of ion and small molecules, which further confirmed the molecular characteristics of the cuproptosis-related lncRNA signature. In GSEA enrichment analyses, the high-risk group tends to enrich in cellular functions such as cell cycle and proliferation, while the low-risk group in biological processes involving energy metabolism. In addition, 28 immune cell infiltration and immune-related function were also analysed via ssGSEA algorithms to elucidate the relationship between this risk signature and the immune microenvironment of tumour. The content of activated, memory, and immature B cell were elevated in the low-risk group. B cells, as vital components of the adaptive immune system, are commonly found in various tumour tissues, such as breast, cervical, and ovarian cancer, and non-small cell lung cancer [34]. It not only plays immune-regulatory function of antibody and antibody-antigen complexes, but also has influence on the functions of other immune or tumour cells via presenting antigens, providing co-stimulation, and secreting cytokines [35]. CD4 T cell, called as T helper (Th) cell, include Th1, Th2, Treg, and Th17 cell. Th cells present highly heterogeneous and several subgroups of them retain synergy in immune regulation and homeostasis maintenance. In the low-risk group, its proportion is significantly higher than that in the high-risk group, indicating that Th cells are essential in tumour growth and progression. Moreover, CD8 T cells decreased markedly in PCa tissues compared to the normal epithelium [36]. Previous studies found that dendritic cell participate in anti-toumur responses against PCa and closely associated with the favourable prognosis in PCa [37]. Natural killer cell activity was negatively associated with the clinical outcomes on prostate biopsy [38]. Meanwhile, the result of immune function shows that the APC co-stimulation, CCR, inflammation-promoting, MHC class I, parainflammation, and type II IFN reponse was down-regulated in the high-risk group. APC co-stimulation is critical in promoting Th cells differentiation as well as initiating and maintaining the immune response [39, 40]. Several chemokines emerged as essential mediators in PCa invasion and metastasis as they do in inflammation [41]. Inflammation is served as a risk factor for PCa [42], and is also related with aggressive disease [43, 44]. Then, the type II IFN response not only promotes immune responses to microorganisms, but also participates in cancer immunosurveillance [45]. What is more, most key immune checkpoints including PD-L1 and PD-L2 are in a low expression level in the high-risk group. Collectively, these findings indicated that the high-risk group is in an immunosuppressive microenvironment, which further leads to a poorer prognosis.

With the development of next-generation sequencing, germline and somatic genetic testing are considered to guide the clinical practice. Based on the identification of DNA repair gene defects, poly (ADP-ribose) polymerase (PARP) inhibitors were developed and utilized to control tumour. Currently, Olaparib was the first PARP inhibitor to show significant activity in metastatic CRPC with prior progression to standard therapy [46]. In a single-arm phase II clinical study, Olaparib had a 50% observed objective response rate and a 60% disease control rate in advanced cancer patients with germline BRCA1/2 mutation [47]. And Konstantinopoulos P et al. reported that there was in vivo synergism between the heat shock protein (HSP) 90 inhibitor AT13387 Onalespib and the PARP inhibitor Olaparib [48]. These are encouraging outcomes. Based on that, the association between the gene mutation and the cuproptosis-related lncRNA signature is evaluated. According to the landscape of somatic gene mutation (Fig. 10A-B), the gene mutation spectrum between both risk groups exist disparity to some extent, indicating that its different may explain or lead to the distinct prognostic outcomes. In addition, TMB and MSI were commonly applied to predict the response of immunotherapy. This result showed that the TMB and MSI score in the high-risk group is significantly higher than that in the low-risk group. Wang et al. found that the high-TMB group had lower overall survival than the low-TMB group [26], which is consistent with the results of this study. Then, the drug sensitivity analysis was also performed via the ‘*pRRophetic*’ package, the results of which demonstrate that patients in the high-risk group benefit more from bicalutamide, while in the low-risk group from docetaxel.

Although this study develops the novel cuproptosis-related lncRNA signature, several limitations should be realized. Fistly, the prognosis signature cannot be validated by external validation cohorts, as other current datasets a lack for the lncRNA sequencing data and complete clinical information. Secondly, the latent mechanism underlying cuproptosis-related lncRNA along with its relationship with tumour immune microenvironment, gene mutation, TMB, MSI, and the drug sensitivity should be further validated in vivo and in vitro experiments.

## Conclusions

In this study, a novel cuproptosis-related lncRNA signature was constructed via machine learning algorithms, along with a nomogram developed based on it and other available common clinical traits, both of which can accurately and reliably predict the DFS of PCa. The prognosis signature was closely associated with several common clinical traits, immune cell infiltration, immune-related functions, immune checkpoints, gene mutation, TMB, MSI, and drug sensitivity, which may be useful to improve the precision treatment and clinical outcome of PCa patients.

## Supplementary Information

**Additional file 1: Supplementary Table S1.** Cuproptosis-related gene from previous literatures. **Supplementary Table S2.** The metagenes list of pan-cancer immune. **Supplementary Table S3.** The primer sequences of β-actin, AC005790.1, AC011472.4, AC099791.2, AC144450.1, LIPE-AS1, and STPG3-AS1. **Supplementary Table S4.** Identification of cuproptosis‑related lncRNAs.

## Data Availability

The datasets generated and/or analyzed during the current study are available in the TCGA repository, (https://portal.gdc.cancer.gov/) and cBioPortal for cancer genomics repository, (https://www.cbioportal.org/).

## Abbreviations

APC: Antigen presenting cell
AR: Androgen receptor
AR-V7: Androgen receptor splice variant 7
AUCs: Areas under the ROC curves
CCR: C–C chemokines receptors
C-index: Harrell’s concordance index
CRPC: Castration-resistant prostate carcinoma
DFS: Disease-free survival
FC: Fold change
Fe-S: Iron-sulfur
GSEA: Gene set enrichment analysis
HSP: Heat shock protein
IC50: Half-maximal inhibitory concentration
IFN: Interferon
K-M: Kaplan–Meier
Lasso: Least absolute shrinkage and selection operator
lncRNA: Long non-coding RNA
m^6^A: N6-methyladenosine
MDSC: Myeloid-derived suppressor cell
MHC: Major histocompatibility complex
MMR: Mismatch repair
MSI: Microsatellite instability
PARP: Poly (ADP-ribose) polymerase
PCa: Prostate carcinoma
PSA: Prostate-specific antigen
RT-qPCR: Real-time quantitative polymerase chain reaction
ROC: Receiver operating characteristic
ssGSEA: Single sample gene set enrichment analysis
TCA: Tricarboxylic acid
TCGA: The cancer genome atlas
TMB: Tumour mutational burden
VEGF: Vascular endothelial growth factor

## References

[1] Siegel RL, Miller KD, Fuchs HE, Jemal A (2022). Cancer statistics, 2022. CA Cancer J Clin.

[2] Sharifi N, Gulley JL, Dahut WL (2005). Androgen deprivation therapy for prostate cancer. JAMA.

[3] Attard G, Murphy L, Clarke NW, Cross W, Jones RJ, Parker CC (2022). Abiraterone acetate and prednisolone with or without enzalutamide for high-risk non-metastatic prostate cancer: a meta-analysis of primary results from two randomised controlled phase 3 trials of the STAMPEDE platform protocol. Lancet.

[4] Boussios S, Rassy E, Shah S, Ioannidou E, Sheriff M, Pavlidis N (2021). Aberrations of DNA repair pathways in prostate cancer: a cornerstone of precision oncology. Expert Opin Ther Targets.

[5] Mercer TR, Dinger ME, Mattick JS (2009). Long non-coding RNAs: insights into functions. Nat Rev Genet.

[6] Soares JC, Soares AC, Rodrigues VC, Melendez ME, Santos AC, Faria EF (2019). Detection of the Prostate Cancer Biomarker PCA3 with Electrochemical and Impedance-Based Biosensors. ACS Appl Mater Interfaces.

[7] Hu R, Lu Z (2020). Long non-coding RNA HCP5 promotes prostate cancer cell proliferation by acting as the sponge of miR-4656 to modulate CEMIP expression. Oncol Rep.

[8] Kim BE, Nevitt T, Thiele DJ (2008). Mechanisms for copper acquisition, distribution and regulation. Nat Chem Biol.

[9] Sproull M, Brechbiel M, Camphausen K (2003). Antiangiogenic therapy through copper chelation. Expert Opin Ther Targets.

[10] Gupte A, Mumper RJ (2009). Elevated copper and oxidative stress in cancer cells as a target for cancer treatment. Cancer Treat Rev.

[11] Ioannidou E, Moschetta M, Shah S, Parker JS, Ozturk MA, Pappas-Gogos G (2021). Angiogenesis and Anti-Angiogenic Treatment in Prostate Cancer: Mechanisms of Action and Molecular Targets. Int J Mol Sci.

[12] Kelly WK, Halabi S, Carducci M, George D, Mahoney JF, Stadler WM (2012). Randomized, double-blind, placebo-controlled phase III trial comparing docetaxel and prednisone with or without bevacizumab in men with metastatic castration-resistant prostate cancer: CALGB 90401. J Clin Oncol.

[13] Piccardo A, Ugolini M, Righi S, Bottoni G, Cistaro A, Paparo F (2020). Copper, PET/CT and prostate cancer: a systematic review of the literature. Q J Nucl Med Mol Imaging.

[14] Tsvetkov P, Coy S, Petrova B, Dreishpoon M, Verma A, Abdusamad M (2022). Copper induces cell death by targeting lipoylated TCA cycle proteins. Science.

[15] Liu C, Gao Y, Ni J, Chen S, Hu Q, Wang C (2022). The ferroptosis-related long non-coding RNAs signature predicts biochemical recurrence and immune cell infiltration in prostate cancer. BMC Cancer.

[16] Zhang Y, Guo S, Wang S, Li X, Hou D, Li H (2021). LncRNA OIP5-AS1 inhibits ferroptosis in prostate cancer with long-term cadmium exposure through miR-128-3p/SLC7A11 signaling. Ecotoxicol Environ Saf.

[17] Liu J, Zhang W, Wang J, Lv Z, Xia H, Zhang Z, et al. Construction and validation of N6-methyladenosine long non-coding RNAs signature of prognostic value for early biochemical recurrence of prostate cancer. J Cancer Res Clin Oncol. 2022. 10.1007/s00432-022-04040-y PMC11798187 35731271

[18] Wen S, Wei Y, Zen C, Xiong W, Niu Y, Zhao Y (2020). Long non-coding RNA NEAT1 promotes bone metastasis of prostate cancer through N6-methyladenosine. Mol Cancer.

[19] Zhou Y, Zhou B, Pache L, Chang M, Khodabakhshi AH, Tanaseichuk O (2019). Metascape provides a biologist-oriented resource for the analysis of systems-level datasets. Nat Commun.

[20] Wang C, Zhang Y, Gao WQ (2022). The evolving role of immune cells in prostate cancer. Cancer Lett.

[21] Boussios S, Rassy E, Moschetta M, Ghose A, Adeleke S, Sanchez E (2022). BRCA Mutations in Ovarian and Prostate Cancer: Bench to Bedside. Cancers (Basel).

[22] Sokolova AO, Cheng HH (2020). Genetic Testing in Prostate Cancer. Curr Oncol Rep.

[23] Charoentong P, Finotello F, Angelova M, Mayer C, Efremova M, Rieder D (2017). Pan-cancer Immunogenomic Analyses Reveal Genotype-Immunophenotype Relationships and Predictors of Response to Checkpoint Blockade. Cell Rep.

[24] Zeng D, Ye Z, Shen R, Yu G, Wu J, Xiong Y (2021). IOBR: Multi-Omics Immuno-Oncology Biological Research to Decode Tumor Microenvironment and Signatures. Front Immunol.

[25] Shah S, Rachmat R, Enyioma S, Ghose A, Revythis A, Boussios S (2021). BRCA Mutations in Prostate Cancer: Assessment, Implications and Treatment Considerations. Int J Mol Sci.

[26] Wang L, Pan S, Zhu B, Yu Z, Wang W (2021). Comprehensive analysis of tumour mutational burden and its clinical significance in prostate cancer. BMC Urol.

[27] Abida W, Cheng ML, Armenia J, Middha S, Autio KA, Vargas HA (2019). Analysis of the Prevalence of Microsatellite Instability in Prostate Cancer and Response to Immune Checkpoint Blockade. JAMA Oncol.

[28] Geeleher P, Cox N, Huang RS (2014). pRRophetic: an R package for prediction of clinical chemotherapeutic response from tumor gene expression levels. PLoS ONE.

[29] Chi Y, Wang D, Wang J, Yu W, Yang J. Long Non-Coding RNA in the Pathogenesis of Cancers. Cells. 2019;8(9):1015. 10.3390/cells8091015 PMC6770362 31480503

[30] Shanbhag VC, Gudekar N, Jasmer K, Papageorgiou C, Singh K, Petris MJ (2021). Copper metabolism as a unique vulnerability in cancer. Biochim Biophys Acta Mol Cell Res.

[31] Saxby H, Mikropoulos C, Boussios S (2020). An Update on the Prognostic and Predictive Serum Biomarkers in Metastatic Prostate Cancer. Diagnostics (Basel).

[32] Chu Y, Liu Z, Liu J, Yu L, Zhang D, Pei F (2020). Characterization of lncRNA-Perturbed TLR-Signaling Network Identifies Novel lncRNA Prognostic Biomarkers in Colorectal Cancer. Front Cell Dev Biol.

[33] Liu J, Liu Y, Gao F, Zhang J, Pan J, Liu Y (2021). Comprehensive study of a novel immune-related lncRNA for prognosis and drug treatment of cervical squamous cell carcinoma. Am J Transl Res.

[34] Burger JA, Wiestner A (2018). Targeting B cell receptor signalling in cancer: preclinical and clinical advances. Nat Rev Cancer.

[35] Tsou P, Katayama H, Ostrin EJ, Hanash SM (2016). The Emerging Role of B Cells in Tumor Immunity. Cancer Res.

[36] Gannot G, Richardson AM, Rodriguez-Canales J, Pinto PA, Merino MJ, Chuaqui RF (2011). Decrease in CD8+ lymphocyte number and altered cytokine profile in human prostate cancer. Am J Cancer Res.

[37] Fridman WH, Remark R, Goc J, Giraldo NA, Becht E, Hammond SA (2014). The immune microenvironment: a major player in human cancers. Int Arch Allergy Immunol.

[38] Barkin J, Rodriguez-Suarez R, Betito K (2017). Association between natural killer cell activity and prostate cancer: a pilot study. Can J Urol.

[39] Reiner SL (2007). Development in motion: helper T cells at work. Cell.

[40] Gallucci S, Matzinger P (2001). Danger signals: SOS to the immune system. Curr Opin Immunol.

[41] Wang JM, Deng X, Gong W, Su S (1998). Chemokines and their role in tumor growth and metastasis. J Immunol Methods.

[42] De Marzo AM, Platz EA, Sutcliffe S, Xu J, Grönberg H, Drake CG (2007). Inflammation in prostate carcinogenesis. Nat Rev Cancer.

[43] Gurel B, Lucia MS, Thompson IM, Goodman PJ, Tangen CM, Kristal AR (2014). Chronic inflammation in benign prostate tissue is associated with high-grade prostate cancer in the placebo arm of the prostate cancer prevention trial. Cancer Epidemiol Biomarkers Prev.

[44] Klink JC, Bañez LL, Gerber L, Lark A, Vollmer RT, Freedland SJ (2013). Intratumoral inflammation is associated with more aggressive prostate cancer. World J Urol.

[45] Dunn GP, Koebel CM, Schreiber RD (2006). Interferons, immunity and cancer immunoediting. Nat Rev Immunol.

[46] Ghose A, Moschetta M, Pappas-Gogos G, Sheriff M, Boussios S (2021). Genetic Aberrations of DNA Repair Pathways in Prostate Cancer: Translation to the Clinic. Int J Mol Sci.

[47] Kaufman B, Shapira-Frommer R, Schmutzler RK, Audeh MW, Friedlander M, Balmaña J (2015). Olaparib monotherapy in patients with advanced cancer and a germline BRCA1/2 mutation. J Clin Oncol.

[48] Konstantinopoulos P, Palakurthi S, Zeng Q, Zhou S, Liu JF, Ivanova E (2016). In vivo synergism between PARP-inhibitor olaparib and HSP90-inhibitor AT13387 in high grade serous ovarian cancer patient derived xenografts. J Clin Oncol.

